# A realist review of the contribution of person-centred cultures to the management of depression among older persons in nursing homes

**DOI:** 10.1016/j.ijnsa.2026.100618

**Published:** 2026-07-03

**Authors:** Tope Omisore, Seán Paul Teeling, Timmy Frawley

**Affiliations:** a University College Dublin School of Nursing, Midwifery, and Health Systems, Dublin, Leinster, Ireland; b Centre for Person-Centered Practice Research, Division of Nursing, Queen Margaret University, School of Health Sciences, Edinburgh, Scotland, UK; c Centre for Interdisciplinary Research, Education and Innovation in Health Systems, University College Dublin, Dublin, Ireland

**Keywords:** Person-centred cultures, Depression, Older persons, Nursing home, Realist review

## Abstract

**Background** Person-centeredness is espoused in many national and international policies and standards and are viewed to inform cultures of care in nursing homes and influence mental health of older adults. In spite of this, studies have shown mixed results regarding the impacts of person-centred cultures on depression with outcomes depending on contexts and implementation approach. In addition, the prevalence of depression in nursing homes remains high. **Objective** This review aimed to understand how, why and in what contexts person-centred cultures, the expected cultures of care in nursing homes, contribute or not to the management of depression among older adults using a realist review. **Method** Realist review is a theory-driven review which explains whether or not an intervention works, how, why and in what contexts, in forms of theories. The theories are in forms of contexts, mechanism, and outcome configurations - CMOCs. This review followed four -step design by Pawson and colleagues and was informed by four initial programme theories. This is a phase two of a wider study. A systematic search of eight data bases including grey literature was conducted to gather evidence to refine the initial programme theories in collaboration with expert and local reference panels. **Results** Forty context, mechanism and outcome configurations derived from thirty relevant and rich papers were theorized, generating demi-regularities which informed the refinement/development of six programme theories. The programme theories highlight: the importance of staff education and leadership support for person-centred care. (ii) the importance of supporting older persons’ autonomy through care planning and communication. (iii) supporting social connection and engagement for older persons through staff relationships. (iv) promotion of independence for older persons through environmental and relational support. (v) the importance of organisational enablers for sustainable person-centred cultures. (vi) the importance of environmental and organisational designs for social and psychological wellbeing of older persons. These show that person-centred cultures can mitigate against depression through individual (e.g. staff’s confidence and competence), relational (trust, mutual familiarity) and organisational mechanisms. **Conclusion** This realist review highlights that person-centred cultures do contribute to the management of depression among older persons in nursing homes. These findings provide actionable insights into how organisational, relational and individual mechanisms interact within nursing homes to support the mental health of older persons. This study was prospectively registered with PROSPERO, ID CRD42024568251 on July 11, 2024

**What is already known**

• Person-centred cultures are viewed as cultures of care in the nursing homes and are viewed to have potential to contribute to positive mental health outcomes for older persons.
• Studies have shown mixed results regarding the impacts of person-centred cultures on depression with outcomes depending on contexts and models of person-centeredness.
• The prevalence of depression among older persons in nursing homes remains high.

Alt-text: Unlabelled box dummy alt text

**What this paper adds**

• Practical explanations in forms of how, why and in what contexts person-centred cultures contribute to the management of depression.
• Highlights the importance of education and training of staff at all levels, the importance of organisational support, environmental adaptations and transformational leadership for person-centred cultures in nursing homes.
• Provide individual, relational and organisational mechanisms that are related to the management of depression among older persons in nursing homes.

Alt-text: Unlabelled box dummy alt text

## 1 Introduction

Depression is a recognised public health concern (Lancet, 2022). Although prevalent among older persons generally, its prevalence is considerably higher among those residing in nursing homes, irrespective of cognitive status (Tesky et al., 2019; Wang et al., 2024), highlighting the burden of depression among older persons in nursing homes. Furthermore, depression has been reported to have negative impacts on the quality of life of older persons it is associated with suicide, reduced self-care abilities, cognitive impairment and increased utilisation of acute care services (Zhang et al., 2018; Murphy et al., 2015; Jain et al., 2022; Wicke et al., 2022).

While depression is reported to have many causes and is associated with many factors ranging from reduced functional abilities, cognitive impairment and the presence of multi-morbidity (Fornaro et al., 2020; Zenebe et al., 2021), the importance of cultures in the nursing homes in relation to the mental health of the older persons has been documented, and organisational cultures generally are associated with patients’ outcomes (American Geriatrics Society, 2003; Cassie and Cassie, 2012; Churruca et al., 2023). For example, Cassie and Cassie (2012) reported that nursing home with proficient cultures where staff responded to the needs of older persons timely, older persons experienced less of depressive symptoms over time.

Different screening instruments and diagnostic criteria are used to assess depression among older persons. Commonly used screening tools include the Geriatric Depression Scale (Poyraz and Bruk Oy, 2025) and the Cornell Depression Scale (Resnick et al., 2022) for older adults living with dementia, while diagnostic criteria such as the Diagnostic and Statistical Manual, 5th edition are also used to establish clinical diagnosis of depression (Devita et al., 2022).

Table 1 gives a comparative prevalence of depression among community dwelling older persons and depression among older persons living in the nursing homes.

**Table 1:** Prevalence rates of depression among older persons in nursing homes and community settings.

Depression classification	Nursing home prevalence	Community-dwelling prevalence
Major depressive disorder (without dementia)	18.9% (Fornaro et al., 2020): A systematic review covering populations of older adults from many countries.	2.1% (Sjöberg et al., 2017)- Swedish population-based study.
Depressive symptoms (without dementia)	39% (Hu et al., 2018)- a national study in Taiwan	9.2-10.6 (Sjöberg et al., 2017) - Swedish population-based study
Major depressive disorder (with dementia)	10.6% (95% CI, 3.4%-33.3%) (Asmer et al., 2018) - a systematic review.	20.0% (95% CI, 8.3%-48.0%) (Asmer et al., 2018)- a systematic review.
Depressive symptoms (with dementia)	46.4% (van Asch et al., 2013) -a Danish cross-sectional study involving about 1900 older adults.	>29% (Kwon and Lee, 2021) - a systematic review.

Footnote. *Prevalence estimates are drawn from the cited studies. Variability reflects differences in study populations, diagnostic criteria, and measurement tools used to assess depression and depressive symptoms.*

Person-centred cultures, conceptualised as a set of care practices and organisational attributes (McCormack, 2022) that promote independence, social engagement, dignity, autonomy and preferences of individuals, are gold standards of care in nursing homes globally and are espoused in many national and international policies and standards (World Health Organisation 2015; Edgar et al., 2020). They are viewed to promote mental health and wellbeing (Carcavilla-González et al., 2025). However, studies of impacts of person-centred care on depressive symptoms have been mixed (Ballard et al., 2018, Brownie and Nancarrow, 2013, Carcavilla-González et al., 2025, Kim and Park, 2017;Chenoweth et al., 2019) and the prevalence of depression in this population remains high (Wang et al., 2024). These variations are often attributed to differences in implementation fidelity, local contextual factors, and the particular model of person-centred care employed. Notably, the majority of existing research has been conducted with populations living with dementia (Brownie and Nancarrow, 2013; Kim and Park, 2017; Ballard et al., 2018; Berkovic et al., 2023, Chenoweth et al., 2019;) and has been outcome-based, meaning the effects of person-centred care on older persons without dementia remain underexplored. Hence, the need for this study which focussed on whether or not person-centred cultures impact on depression among older adults (with or without cognitive impairment) in nursing homes, how, why and in what contexts. While the effects of person-centred care on older persons without dementia are underexplored, the current study focussed on both populations of older adults living with dementia and those who are not.

Studying complex healthcare interventions such as the person-centred cultures requires a methodology which can unpack the complexity of both healthcare intervention and health systems (Wong, 2018). Unlike conventional systematic reviews, realist reviews are theory-driven reviews which explain and not just give a judgement about whether an intervention works (Wong, 2018). Realist reviews answer research questions about whether an intervention works, how, why, for whom, and in what contexts (Hunter et al., 2022), thereby unpacking the contextual complexity that characterises healthcare settings (Wong, 2018). Realist review aims to develop, test and refine programme theories, thereby starting and ending with theories containing contexts, mechanism and outcome configurations (CMOCs) (Hunter et al., 2022). A programme theory is an explanation of how and why an intervention brings about a change (Pawson, 2006). Contexts are personal, interpersonal, institutional or infrastructural factors that enable or hinder mechanism from being generated (Pawson, 2006). Mechanism is a process generated by a specific context (Pawson, 2006). Mechanisms’ constructs include resources and reasoning (Pawson, 2006), forces, interaction, processes, powers and liabilities (Westhorp, 2018) and are viewed to bring about a change (outcome). The outcome which may be intended or unintended change (Pawson, 2006), in this current study, which is the management of depressive symptoms, includes prevention and reduction of depressive symptoms while also including broader psychological wellbeing outcomes associated with person-centred cultures such as autonomy.

The involvement of expert and local reference panels adds another advantage to usefulness of realist review by ensuring relevance and transferability of findings (Shé et al., 2018). Realist review aims to enhance policy or intervention implementation through transfer of mechanism and has been used as a research methodology in healthcare related research (Halsall et al., 2022, Teeling et al., 2021).

Different terms and classifications are used in the literature to describe nursing homes, reflecting variations in healthcare systems, levels of care provision, and organisational structures across countries (Sanford et al., 2015). Please, see Table 2 for different terms and definitions used internationally. Sanford and colleagues advised that it is imperative to clearly operationalise the definition of nursing home in any research endeavour. Therefore, for the purpose of this review, a nursing home is operationalised as a long-term care facility where an older adult irrespective of cognitive status, receives nursing care in addition to assistance with activities of daily living for an indefinite period of time (Froggatt et al., 2013). The review therefore included facilities accommodating older adults with and without dementia, including dementia-specific units located within nursing homes or long-term care facilities. Facilities were included where nursing care formed a core component of service provision and where residents required ongoing support with activities of daily living and health-related needs. Consequently, the review included studies conducted in community nursing units, residential aged care facilities with nursing provision, skilled nursing facilities, and dementia care units within nursing homes where applicable.

**Table 2:** Terms and definitions of nursing homes internationally (Adapted from Sanford et al., 2015).

Term	Definition
Hospice home	Provides end-of-life care for older adults.
Geriatric hospital	Provides transitional care following an acute hospital admission.
Care home	Provides assistance with activities of daily living (ADLs) without licensed healthcare professionals on site.
Assisted living facility	Provides accommodation and support with activities of daily living (ADLs).
Skilled nursing facility	Provides short-term skilled care, typically following discharge from an acute hospital.
Rehabilitation hospital	Provides physiotherapy, occupational therapy, and speech and language therapy to restore or maximise function.
Long-term care facility	Provides accommodation and assistance with ADLs; may include ongoing management of chronic conditions.
Subacute care facility	Provides step-down care following hospitalisation, including rehabilitation therapies.
Community Nursing Unit (CNU)	Irish term for modernised public nursing homes providing 24-hour care for older adults, with an emphasis on person-centred environments.

**Fig. 1:**
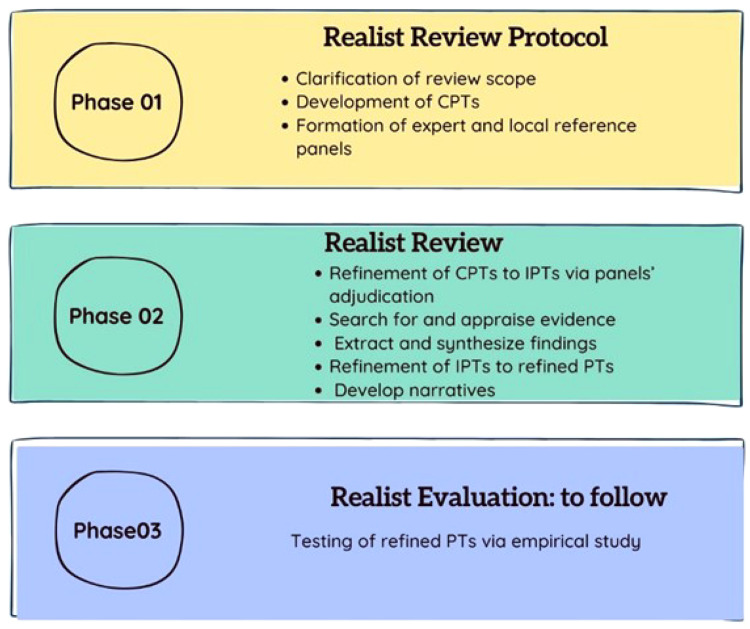
Overview of study, adapted from (Omisore et al., 2025): CPTs - candidate programme theories, IPTs- initial programme theories, PTs- Programme theories.

This realist review is a part of a wider doctoral study which seeks to explore the contribution of person-centred cultures to the management of depression among older persons in community nursing units in Ireland using realist inquiry. The study is divided into three phases (Fig. 1).

Phase one of the study has been detailed in a published protocol paper (Omisore et al., 2025). This current paper details phase two of the study which is the realist review, that aimed to evaluate what about person-centred cultures contributes/does not contribute to the management of depression among older persons in nursing homes, for whom, in what context, how and why? The third phase of the study, a realist evaluation, will take place in study sites in Ireland in 2026.

## 2 Methods

### 2.1 Design

Realist review is a methodological choice for this study because it has been used for evaluating complex interventions involving person-centeredness (Wong, 2018) and for unpacking contextual complexities that characterise healthcare settings such as nursing homes (Teeling et al., 2021). The review was prospectively registered with the International Prospective Register of Systematic Reviews (CRD42024568251) and the protocol has been published (Omisore et al., 2025). The aim of this review was to gather from available evidence (published and unpublished) and the grey literature if and how person-centred cultures contribute or do not contribute to the management of depression among older persons (with or without cognitive impairment) in nursing homes using realist review. The research questions that guided the review are as follow:

• Do person-centred cultures contribute (or not) to the management of depression among older persons in nursing homes, how, for whom, in what contexts and why?
• What are the facilitators and barriers of developing person-centred cultures that may contribute to the management of depression (prevention and reduction of depressive symptoms and psychological wellbeing) among older persons in nursing homes?
• In what contexts do specific mechanisms facilitate or hinder nursing home staff’s understanding of the development of person-centred cultures, and how does this understanding influence the management of depressive symptoms among older persons?

The Realist and MEta-narrative Evidence Syntheses: Evolving Standards (RAMESES I) (Wong et al., 2013) guided the reporting of the review. The completed RAMESES checklist is presented in appendix A. The review followed the four-step design by Pawson and colleagues (Pawson et al., 2005) as follows:

• Define the scope of the review,
• Search for and appraise evidence,
• Extract and synthesize findings,
• Develop narratives.

#### 2.1.1 Define the scope of review

Collaborating with a local reference group and an expert panel is consistent with realist research (Shé et al., 2018). A local reference group and an expert panel were convened in January 2024 to clarify the scope of the review and ensure the relevance and practicality of the developing programme theories (Teeling et al., 2021). The local reference group (LRG) consisted of staff working in the nursing home sector. The expert panel comprised nurses, academics, and researchers from Ireland, Sweden, and Australia with expertise in person-centred practice, mental health, realist methodology, and the care of older persons, including care within nursing home settings. The multidisciplinary composition of the panel provided a breadth of clinical, methodological, and theoretical perspectives relevant to the review topic. The expert panel contributed to several stages of the review process. In collaboration with the research team, panel members assisted in refining the review question, clarifying the scope and purpose of the review, and ensuring the review focus on person-centred cultures and depression among older persons in nursing homes and also signposted to relevant literature. The panel also contributed to the interpretation of the context–mechanism–outcome (CMO) configurations and programme theories refinement. In particular, the expert panel reviewed the developing programme theories for conceptual relevance, coherence, and consistency with person-centred practice and older persons’ care. Their input strengthened the credibility and theoretical robustness of the review findings. Eight candidate programme theories (rough explanations of how contexts and mechanism interact to generate outcomes) were then developed through consultation with the local reference group, informed by seminal literature (Frijters et al., 2011; Kitwood, 1997; McCormack et al., 2010; Sabat, 1994; Diegelmann et al., 2018), and through review of relevant regulatory documents, including the Health Information and Quality Authority standards for nursing homes in the Republic of Ireland (Health Information and Quality Authority: National standards for residential care settings for older people in Ireland, 2016). The seminal literature was identified from searches in CINAHL and google scholar using the keywords person-centredness, older persons, nursing home and depression (Teeling et al., 2021). The eight candidate programme theories related to staff training, authentic relationship between staff and older persons, social engagement for older persons, respect for choice and autonomy of older persons, use of care plans to provide individualised care, promotion of meaningful activities and support for independence of older persons, and are available for review in the published realist review protocol (Omisore et al., 2025).

As detailed in the protocol for this review (Omisore et al., 2025), the eight candidate programme theories were brought to the expert panel in May 2024. Through this expert adjudication, candidate programme theories 1 and 5 were refined and became initial programme theories 1 and 2 respectively. Candidate programme theories 2, 3,6 and 8 were amalgamated into initial programme theory 3 because they were deemed by the expert panel as similar.

**Fig. 2:**
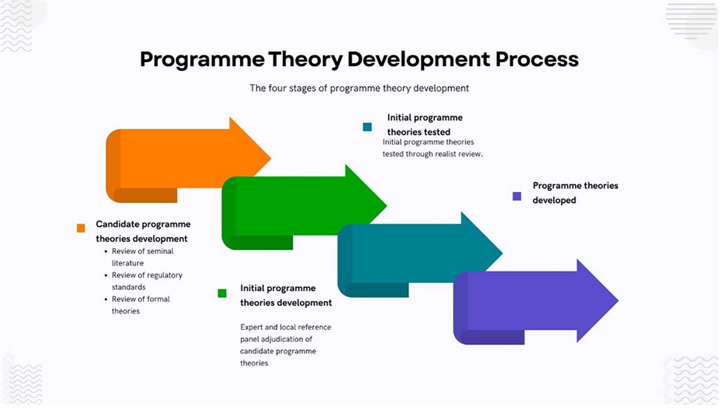
Process of development and refinement of programme theories.

Candidate programme theory 7 was confirmed (became initial programme theory 4, resulting in four agreed initial programme theories (Appendix B). Fig. 2 summarises the development and refinement of programme theories in this review. The resulting four initial programme theories, with the research questions guided the next phase of the review which constitutes phase 2 of the wider study which commenced with the search and appraisal of evidence.

#### 2.1.2 Search for and appraise evidence

This stage of the review was guided by the initial programme theories (Appendix B) and the review question, informing search criteria, inclusion and exclusion of papers and analysis of evidence. Searching and appraisal were refined iteratively as evidence was reviewed (Wong et al., 2013).

##### 2.1.2.1 Data sources

The following databases were searched for published data and grey literature: CINAHL, PubMed/MEDLINE, EMBASE, PsycINFO, Web of Science, Cochrane databases and repositories, and additional literature suggested by the expert panel.

##### 2.1.2.2 Search strategy

A structured search strategy was developed in consultation with an information specialist and informed by the initial programme theories and the review question using the Population-Intervention-Comparison-Outcome (PICO) model (Teeling et al., 2021)—the population here being older persons living in nursing homes, intervention being person-centred cultures, comparison (no comparison was specified in this review) and outcome, prevention and amelioration of depressive symptoms or any proxy outcome as defined above). Search terms combined concepts relating to older persons, nursing homes, person-centred cultures, and depression, and were adapted for each database using controlled vocabulary (e.g., MeSH, CINAHL Headings) and Boolean operators. Searches were limited to studies published from 1990 onwards to reflect the emergence of person-centred care (Kitwood, 1997, Li and Porock, 2014). No methodological filters were applied. The search strategy was pilot tested in PubMed and adapted across other databases. A summary of the search strategy for PubMed is presented in Appendix C.

##### 2.1.2.3 Eligibility criteria

Only studies involving older persons (age 60 and above) in nursing homes (as defined earlier) from 1990 (approximate start of the person-centred movement) (Li and Porock, 2014) were included. Appendix D details inclusion and exclusion /relevance criteria.

##### 2.1.2.4 Data appraisal

Search results were imported into Covidence, and EndNote software was used to record references from search results and additional documents, following the Preferred Reporting Items for Systematic Reviews and Meta-Analyses (PRISMA) guidelines (Page et al., 2021). Rather than using rigid assessment tools for evidence appraisal, which has been described as positivist in nature, evidence appraisal was based on relevance, richness and rigour using the framework of Dada et al. (2023). Relevance refers to a document’s ability to contribute to theory development or refinement; richness refers to its ability to explain the relationship among context, mechanism and outcome (Booth et al., 2013); and rigour refers to the trustworthiness of a document or theory (Booth et al., 2013). Thus, a document may be relevant without being sufficiently rich to be put forward for data extraction. Appendix D details an example of the application of the relevance, richness and rigour criteria for this study. During this review, no document was excluded based on rigour, as methodologically weak documents may nevertheless contain key ideas relevant to theorisation in realist research (Pawson et al., 2005).

##### 2.1.2.5 Screening

Two reviewers (TO and SPT) independently screened titles and abstracts, followed by full texts, for relevance based on the inclusion and exclusion criteria. The third reviewer (TF) acted as an arbitrator when there were disagreements. Documents found to be relevant (n = 65) were also screened independently by two reviewers for richness based on criteria in Appendix D.

#### 2.1.3 Extraction and synthesis of findings

Data extraction was completed by adapting and using the data extraction form developed by Molitor et al. (2023). Extraction was undertaken by the first reviewer (TO) and verified by the second reviewer (SPT). Document characteristics including the title, study objectives, companion papers, study design, richness rating and rigour rating were recorded for each document. The remaining components of the form focused on identifying contexts, mechanisms and outcomes, and their links to the initial programme theories, including any unintended change. Appendix E gives an illustrative example of a completed data extraction form for this study.

Data extraction was completed iteratively using realist logic to identify and interpret context–mechanism–outcome configurations (Aivalli et al., 2025). Each configuration was reviewed against the evidence iteratively by the first reviewer (TO) and verified by the second reviewer (SPT). High- and moderate-richness papers were extracted, while low and no-richness papers were not extracted (Aivalli et al., 2025).

Evidence synthesis involved identifying recurring patterns, or demi-regularities (Aivalli et al., 2025), across related context–mechanism–outcome configurations. These demi-regularities informed the refinement of the initial programme theories and the generation of two additional programme theories. Based on these demi-regularities, aspects of the initial programme theories were confirmed, refined or refuted in alignment with realist research principles (Fletcher, 2017).

#### 2.1.4 Develop narratives

The resulting programme theories were reviewed asynchronously by the local reference group and expert panels offering their insights on the contextual relevance and trustworthiness of the theories generated (Shé et al., 2018). The programme theories were said to be relevant to the lived experience of knowledge users and older persons in the nursing home sector and were said to be coherent.

### 2.2 Ethics

Ethical approval was not required as this study synthesized existing literature.

## 3 Search result

**Fig. 3:**
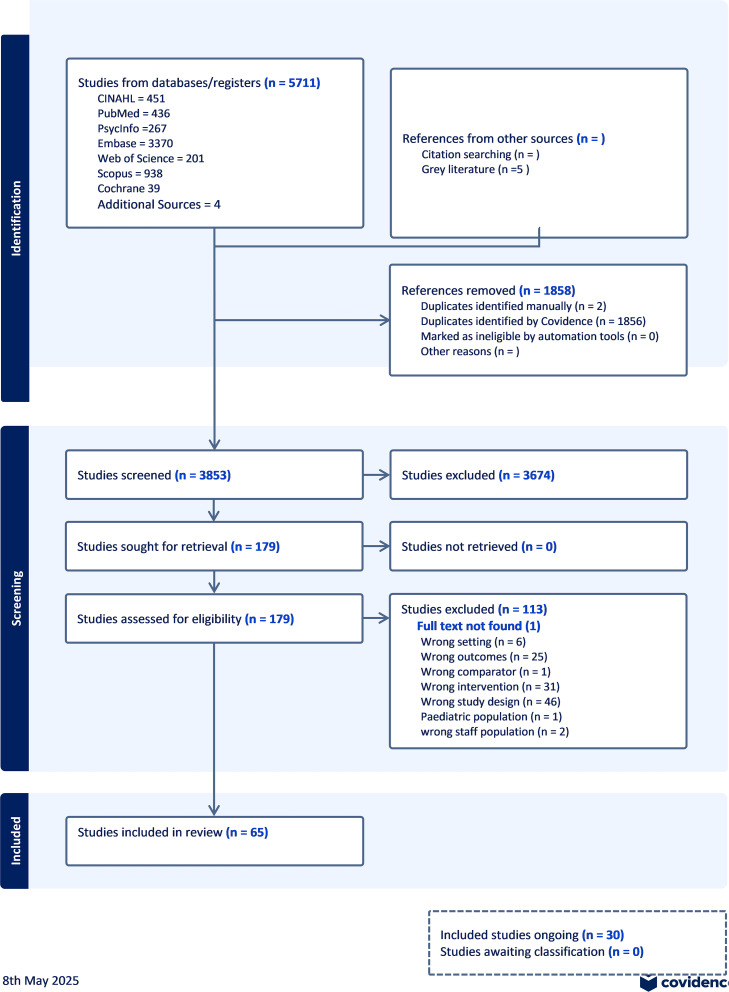
PRISMA Flow chart.

Searches across the data bases run in December of 2024 yielded a total of 5702 results and additional 9 documents from citation searches and from expert panel making a total of 5711 documents. 1859 duplicates were removed resulting into 3853 papers (please see Fig. 3, PRISMA flow chart). which were screened by title and abstract. 179 papers were screened by full text for relevance resulting into 65 papers which were further screened for richness. No full text was found for one paper (Rojano i Luque et al., 2021). 35 low and no- richness papers were not included for data extraction based on their richness levels (Robinson and Rosher, 2006; Crandall et al., 2007; Slettebø, 2008; Davison et al., 2013; Mueller et al., 2013; O'Dwyer, 2013; Schenk et al., 2013; Juk Hyun, 2013; Smith et al., 2013; De Vriendt et al., 2019; Haugan, 2014; Chodosh et al., 2015; Mjørud and Røsvik, 2015; Williams et al., 2015; Donnelly and MacEntee, 2016; Abrams et al., 2017; Travers, 2017; Koopmans et al., 2018; Caspar et al., 2019; Gillis et al., 2019; White et al., 2019; Lee et al., 2020; Reinhardt et al., 2020; Maenhout et al., 2020; Van Haitsma et al., 2020; Chang et al., 2021; McCabe et al., 2021; Oliver et al., 2021; Madrigal et al., 2022; Compton et al., 2022; Ejaz et al., 2022; Midje et al., 2022; Chin and Lee, 2023, van den Eijnde et al., 2024), resulting into thirty high and medium rich papers included in the review from which forty context, mechanism and outcome configurations were extracted. Appendix F gives an illustrative example of the process of analysis and synthesis of findings resulting into the refinement and the development of the initial programme theories into programme theories.

### 3.1 Documents characteristics

The studies included in the review were undertaken in developed countries including Sweden, the United States of America and Canada. Forty percent and about thirty four percent of the studies used qualitative methods and quantitative methods. Other studies used randomised control trials approach, mixed methods and three of the documents included are commentaries and opinion pieces with the central theme of the included papers being management of depression among older persons. Some of the included studies employed the use of tools such as the Geriatric Depression Scale (GDS (Poyraz and Bruk Oy, 2025)) and Cornell Scale for Depression in Dementia (CSDD) (Resnick et al., 2022) to identify depressive symptoms. All the papers were assessed for relevance, richness and rigor and causality established via triangulation (Aivalli et al., 2025). Appendix G contains the summaries of the included studies and components of person-centred cultures relevant to the programme theories.

### 3.2 Developing programme theories

**Fig. 4:**
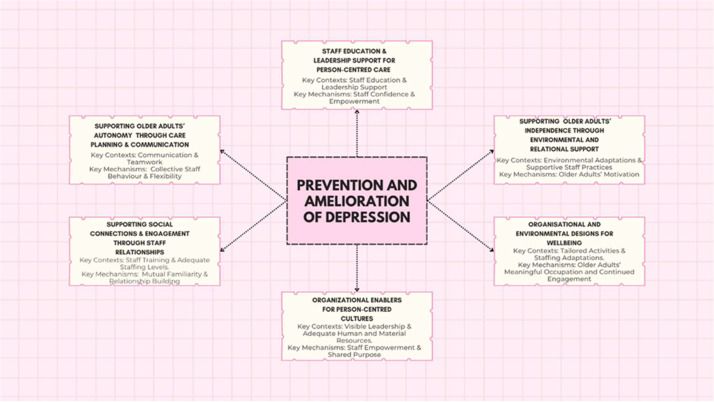
Conceptual framework of programme theories of the person-centred management of depression among older adults in nursing homes.

Source: Authors.

Using a realist analytic approach (Aivalli et al., 2025), recurring demi-regularities across forty context–mechanism–outcome configurations informed iterative refinement of the initial programme theories. This process both sharpened the scope of the four initial programme theories and generated two additional programme theories not fully represented in the original set, resulting in six final programme theories (Fig. 4). The programme theories are outlined in the findings.

## 4 Findings

### 4.1 Programme theories

#### 4.1.1 Programme theory one

**Staff Education and Leadership Support for Person-centred Care**

*In nursing homes where staff at all levels are educated and trained in person-centred approaches including care for depression and there are identifiable person-centred practice champions or practitioner-leaders who support a culture of continuous development and where staff workload is manageable, the mechanisms of leadership support (including mentorship, motivation and role modelling), along with staff confidence, motivation, innovation and empowerment to adapt to older persons’ changing needs result in a culture of embedded person-centred practices, stronger staff–older person relationships and communication, staff taking older persons’ needs seriously, upholding autonomy, and facilitating early detection and amelioration of depressive symptoms.*

This programme theory was refined from Initial Programme Theory One and is supported by five context–mechanism–outcome configurations (CMOs 1, 2, 8, 16, and 19) across four documents (Chenoweth et al., 2014, Wright, 2010; Ho et al., 2021; Schweighart et al., 2022). Across these contexts, mechanisms and outcome configurations, a recurring demi-regularity was identified: education and training in person-centred approaches were most effective when provided to staff at all organisational levels, rather than being limited to direct care roles. Evidence consistently indicated that organisation-wide education enhanced staff confidence, motivation, and responsiveness to older persons’ needs, enabling more adaptive person-centred care practices (Ho et al., 2021, Wright, 2010). These mechanisms were associated with improved autonomy for older persons and with the early detection and amelioration of depressive symptoms (Wright, 2010; Chenoweth et al., 2014, Ho et al., 2021; Schweighart et al., 2022). The identification of this pattern informed refinement of the initial theory to Programme Theory One.

#### 4.1.2 Programme theory two

**Supporting Older Persons’ Autonomy through Care Planning and Communication**

*In nursing homes where there is a culture of respect for older persons’ choice and autonomy, where staff work in teams and involve families in developing individualised care plans, where staff are trained in cultural competence and value diversity/person-centred practices, and where effective communication with families and within teams is prioritised, and where there are adequate supports and resources for this culture, mechanisms such as person-centred care plans based on periodic assessment and family input facilitate staff understanding of older persons and collective staff behaviour to meet older persons’ needs. Staff are empowered to provide individualised care based on care plans that legitimise and facilitate continuity of care, while cultural competence fosters coordinated and consistent care. Perceived availability of support and resources enables older persons to express their choices and staff to be flexible in meeting needs. As a result, older persons experience enhanced autonomy, dignity, meaningful engagement with life, and reduced depressive symptoms.*

This programme theory was refined from Initial Programme Theory Two and is supported by eight contexts-mechanisms-outcomes configurations (8 CMOs) across eight documents (Ho et al., 2021; Cohen-Mansfield and Parpura-Gill, 2008; Backman et al., 2020; Xiao et al., 2023; Duan et al., 2020; Erlandsson et al., 2023; Backman et al., 2024, Duan et al., 2024). Findings demonstrated that person-centred care plans functioned as a key mechanism by facilitating staff understanding of older persons and enabling coordinated, collective staff responses (Ho et al., 2021; Backman et al., 2020; Duan et al., 2020; Xiao et al., 2023). Training in person-centred practice and cultural competence further strengthened staff confidence and consistency in care delivery (Cohen-Mansfield and Parpura-Gill, 2008; Xiao et al., 2023; Backman et al., 2024). Organisational commitment and resourcing were repeatedly identified as enabling contexts that supported staff flexibility and older persons’ willingness to express preferences (Cohen-Mansfield and Parpura-Gill, 2008; Duan et al., 2020, Erlandsson et al., 2023). These mechanisms were associated with enhanced autonomy, dignity, meaningful engagement, and reduced depressive symptoms (Duan et al., 2020, Duan et al., 2024 informing refinement of the initial theory.

#### 4.1.3 Programme theory three

**Supporting Social Connection and Engagement through Staff Relationships**

*In nursing homes where there is a culture of promoting social engagement, where staff know older persons well and understand their values, preferences, and histories, where staff are trained in cultural competence and person-centred communication, and where there are adequate staffing levels and low staff turnover, mechanisms of mutual familiarity and relationship-building, staff confidence, staff empowerment, cultural competence, and supportive attitudes towards participation enable older persons to experience meaningful engagement, autonomy, self-esteem, and meaningful relationships, leading to reduced or prevented depressive symptoms.*

This programme theory is supported by nine contexts-mechanisms-outcomes configurations ( 9 CMOs) across nine documents (Carcavilla-González et al., 2025; Wright, 2010; Chenoweth et al., 2014; Xiao et al., 2023; Haugan et al., 2013, Lindsey Jacobs et al., 2019, Lou et al., 2013, Lyne et al., 2006, Potter et al., 2018, Schenell et al., 2020, Yoon, 2018; ; ). Findings consistently showed that staff knowledge of older persons’ values, histories, and preferences enabled meaningful and supportive social engagement (Carcavilla-González et al., 2025; Wright, 2010). Training in person-centred communication and cultural competence enhanced staff confidence and relational competence (Xiao et al., 2023; Schenell et al., 2020, Yoon, 2018). Adequate staffing levels and low staff turnover were repeatedly identified as enabling sustained relationship-building and mutual familiarity (Haugan et al., 2013, Schenell et al., 2020). These mechanisms were associated with enhanced autonomy, self-esteem, social engagement, and the amelioration of depressive symptoms (Haugan et al., 2013, Lou et al., 2013; Schenell et al., 2020; Yoon, 2018; Lyne et al., 2006), informing refinement of Programme Theory Three.

#### 4.1.4 Programme theory four

**Promoting Independence through Environmental and Relational Support**

*In nursing homes that have a culture of promoting independence of older persons, where the environment is designed for accessibility and homeliness, and where staff engage in independence-supportive practices such as encouragement, older persons feel prompted, supported, and motivated to maintain independence. Older persons’ autonomy, self-worth, and social interactions are enhanced; independence and functional abilities are preserved, with amelioration of depressive symptoms.*

This programme theory is supported by seven context-mechanism-outcome configurations (CMOs 4, 19, 22, 24, 33, 34, and 38) across seven documents (Ho et al., 2021; Chenoweth et al., 2014; Schenell et al., 2020; Lindsey Jacobs et al., 2019; Potter et al., 2018; Backman et al., 2021, Sjögren et al., 2013). Findings highlighted the importance of environmental adaptations for accessibility and homeliness as enabling contexts that prompted and supported independence (Chenoweth et al., 2014; Schenell et al., 2020). Independence-supportive staff practices, such as encouragement and facilitation, were also found to motivate older persons to maintain independence, self-worth, and social interaction (Schenell et al., 2020; Lindsey Jacobs et al., 2019). Evidence did not support a distinct role for person-centred champions within this theory. These findings informed refinement of the programme theory to reflect environmental and relational mechanisms more accurately.

#### 4.1.5 Programme theory five

**Organisational Enablers for Sustainable Person-centred Cultures**

*In nursing homes where there are transformational leaders trained in person-centred care, and where the organisation commits to a person-centred culture through supportive policies, adequate resources (including staffing and training opportunities), and a shared vision that rejects ageist attitudes, mechanisms of perceived transformational leadership and perceived organisational support enable staff commitment to the development and sustainability of person-centred cultures. As a result, person-centred cultures become embedded in practice, older persons experience individualised care that upholds autonomy, and depressive symptoms are ameliorated.*

This is a newly generated programme theory supported by eight context-mechanism-outcome configurations across eight documents (Backman et al., 2020; Cohen-Mansfield and Parpura-Gill, 2008; Wikström and Emilsson, 2014; Backman et al., 2021; Murphy, 2007; Kusmaul and Tucker, 2020; Hung et al., 2016; van Loon et al., 2023). Findings demonstrated that visible leadership, organisational commitment, and adequate resourcing were central enabling contexts for sustaining person-centred cultures (Backman et al., 2020; Hung et al., 2016; Kusmaul and Tucker, 2020). When leadership support and organisational infrastructure were perceived by staff, mechanisms of empowerment, commitment, and shared purpose were activated (Backman et al., 2020; Cohen-Mansfield and Parpura-Gill, 2008). These mechanisms were associated with sustained person-centred practices and improved outcomes for older persons, including upheld autonomy and reduced depressive symptoms (Wikström and Emilsson, 2014; Backman et al., 2021; Murphy, 2007; Kusmaul and Tucker, 2020; Hung et al., 2016; van Loon et al., 2023).

#### 4.1.6 Programme theory six

**Environmental and Organisational Design for Social and Psychological Wellbeing**

*In nursing homes where there are tailored activities and environmental adaptations that support mobility, access, and engagement, where there are activity coordinators and resources for social programming, and where leadership facilitates social participation even during restricted periods such as infection outbreaks, mechanisms including meaningful activities, environmental and staffing adaptations, and innovation in maintaining participation enable older persons to remain connected, supported, and engaged. As a result, older persons experience meaningful activity, social and spiritual connection, and prevention or mitigation of depressive symptoms.*

This newly generated programme theory is supported by six contexts, mechanisms and outcome configurations across six documents (Ho et al., 2021; Duan et al., 2024; Knippenberg et al., 2023; Dichter et al., 2020; Lindner et al., 2023; Hunter et al., 2016). Findings indicated that tailored activities and environmental adaptations enabled older persons to feel supported, enhanced autonomy, and promoted social engagement (Knippenberg et al., 2023; Hunter et al., 2016). Incorporation of meaningful activities into daily routines and the presence of activity coordinators were consistently associated with sustained engagement (Ho et al., 2021; Duan et al., 2024). Leadership-supported prioritisation of social participation during infection outbreaks further enabled continued connection and meaningful occupation (Knippenberg et al., 2023; Dichter et al., 2020; Lindner et al., 2023; Hunter et al., 2016). These mechanisms were associated with the prevention or mitigation of depressive symptoms.

The implications of these findings are explored in the following discussion section.

## 5 Discussion

### 5.1 Interpretation of programme theories

The refined programme theories presented in this review build on the initial programme theories developed from candidate programme theories identified at the outset of the review. Through iterative synthesis, the four initial programme theories were refined and consolidated, while two additional programme theories emerged from the evidence, resulting in six interconnected evidence-informed programme theories. Collectively, these theories provide an explanatory account of how person-centred cultures within nursing homes influence the prevention and amelioration of depressive symptoms among older persons.

Programme Theories One and Five emphasise the importance of staff education, leadership, and organisational support in shaping person-centred cultures and mental health outcomes in nursing homes. Education and training extended beyond direct care staff to include all organisational levels, reflecting the view that person-centred cultures depend on shared values and collective commitment rather than isolated practice change. Training in person-centred approaches elicited staff confidence, competence, empowerment, flexibility, and responsiveness, enabling staff to adapt to the complex and evolving needs of older persons. These mechanisms contributed to the embedding of person-centred practices in daily care and supported the early recognition and amelioration of depressive symptoms.

These findings are consistent with the findings of Ho et al. (2021), who found that organisation-wide education fostered staff commitment to person-centred values and enhanced residents’ wellbeing. Similarly, Backman et al. (2020) highlighted the importance of visible and supportive leadership in sustaining person-centred cultures, particularly in contexts where staff workloads are manageable (Schweighart et al., 2022). Together, these findings suggest that education, leadership, and organisational support operate synergistically: education equips staff with the necessary knowledge and skills, while leadership and organisational structures create conditions that enable these capabilities to be enacted and sustained in practice.

The programme theories also resonate with the concept of psychological safety in healthcare settings (Peddie et al., 2025), characterised by staff confidence, perceived support, and reduced burnout (Peddie et al., 2025). Psychological safety appeared to function as an enabling mechanism that allowed staff to engage relationally with residents, exercise professional judgement, and adapt care practices without fear of blame. This is particularly relevant given the high levels of burnout, occupational stress, and workplace violence reported among nursing home staff (Turnpenny, 2021). Organisational investment in leadership, staff development, and supportive workplace cultures may therefore contribute not only to workforce wellbeing but also act as a strategy to improve mental health outcomes for older persons.

Programme Theory Two highlights care planning and communication as key strategies through which person-centred values are translated into practice. Care plans were most effective when developed collaboratively with older persons and families, actively used by staff, and supported by organisational commitment and adequate resources. Under these conditions, care plans functioned not merely as documentation tools but as relational and communicative artefacts that promoted continuity, shared understanding, and individualised care. These processes supported older persons’ autonomy, dignity, and meaningful involvement in care, contributing to improved emotional wellbeing and reduced depressive symptoms.

This interpretation aligns with Self-Determination Theory (Deci and Ryan, 2012), which identifies autonomy as a core psychological need underpinning wellbeing and motivation, and with evidence linking autonomy to reduced depressive symptoms and improved life satisfaction among older adults (Kloos et al., 2018). The findings further suggest that autonomy-supportive care is shaped by collective organisational processes rather than individual staff actions alone. Where staff teams shared understanding of residents’ needs and preferences, and sufficient resources existed to enact care plans, older persons were more likely to express preferences and participate meaningfully in decision-making. This reinforces the view that autonomy in nursing home settings is co-produced through organisational and relational mechanisms.

Programme Theory Three focuses on the role of staff–resident relationships in supporting social connection and emotional wellbeing. Staff training in person-centred communication and cultural competence (Wright, 2010, Xiao et al., 2023), combined with adequate staffing levels, continuity of care, and staff familiarity with residents’ histories and preferences (Carcavilla-González et al., 2025; Wright, 2010; Xiao et al., 2023), created conditions for meaningful relational engagement. These conditions fostered trust, mutual familiarity, autonomy, and social connectedness, contributing to reduced loneliness and depressive symptoms among older persons.

These findings are consistent with socioemotional selectivity theory (Carstensen, 2021), which emphasises the increasing importance of emotionally meaningful relationships in later life, and with broader evidence demonstrating the protective effects of social connection on mental health (Lim et al., 2023). Importantly, the findings illustrate how organisational factors such as staffing levels, turnover, and training influence the quality and sustainability of relational care in nursing home settings. They suggest that social engagement should not be viewed as an optional or ancillary aspect of care, but as a core component of mental health support in nursing homes.

Programme Theories Four and Six highlight the contribution of environmental, organisational, and relational supports to independence, social participation, and psychological wellbeing. Environmental adaptations (Knippenberg et al., 2023), meaningful activities integrated into daily routines (Ho et al., 2021), activity coordinators (Duan et al., 2024), flexible organisational practices, and innovative approaches to maintaining social participation during infectious disease outbreaks (Lindner et al., 2023; Dichter et al., 2020) acted as enabling conditions that supported motivation, meaningful occupation, and continued social and spiritual engagement among older persons. These mechanisms contributed to the prevention and amelioration of depressive symptoms.

These findings align with person–environment fit theory (Zhang et al., 2025), which emphasises the importance of congruence between individuals and their environments in supporting wellbeing, as well as literature demonstrating the influence of the built and social environment on older adults’ mental health (Rautio et al., 2018). The findings further suggest that supportive environments are not static features of care settings but dynamic processes requiring continuous organisational attention, adaptability, and leadership support, particularly during periods of crisis.

The significance of these programme theories is reinforced by longstanding challenges within nursing homes, including staffing shortages (Kirkham and Lesser, 2020), limited leadership support (PHI 2016), vulnerability to infectious disease outbreaks (Gleckman and Favreault, 2021), loneliness among older persons (Victor, 2012), and the persistence of task-oriented care cultures (Dys et al., 2022). These pressures, intensified during the COVID-19 pandemic, highlight the importance of adaptive and innovative approaches such as technology-enabled social engagement, sustained workforce development, enhanced leadership support, and renewed prioritisation of person-centred cultures to promote the social and psychological wellbeing of older persons in nursing homes (Johs-Artisensi et al., 2023; McArthur et al., 2021).

Taken together, the refined programme theories indicate that depression among older persons in nursing homes is relationally, environmentally, and organisationally mediated. Staff psychological wellbeing, emotional labour, and organisational conditions interact to shape relational authenticity, care quality, and older persons’ mental health outcomes. These findings reinforce the importance of organisation-wide adoption of person-centred cultures in long-term care and provide a theoretically informed foundation for future interventions, policy development, and research aimed at improving mental health and wellbeing among older persons in nursing homes.

### 5.2 Contributions to the literature and implications of findings

To the best of the reviewers’ knowledge, this is the first published realist review to examine the relationship between person-centred cultures and depression among older persons in nursing homes. By applying a realist analytic lens, this review extends the existing literature beyond descriptive or outcome-focused accounts of person-centred care by explaining how, why, and in what contexts person-centred cultures influence depressive symptoms among older persons in long-term care settings.

Through realist synthesis, four initial programme theories were refined and consolidated, and two additional programme theories were generated, resulting in six evidence-driven programme theories. Collectively, these theories advance current knowledge by explicating the mechanisms through which person-centred cultures operate, rather than conceptualising person-centred care as a uniform or static intervention. In particular, this review theorises staff-related processes—such as confidence, empowerment, motivation, responsiveness, commitment, collective behaviour, and flexibility—as mechanisms, rather than individual attributes, through which person-centred cultures contribute to the amelioration of depressive symptoms among older persons in nursing homes.

This review also contributes to theory development by building on and refining existing conceptual frameworks, including the Person-Centred Nursing Framework (McCormack and McCance, 2021) and Person–Environment Fit Theory (Zhang et al., 2025).

With respect to the Person-Centred Nursing Framework, the findings extend existing conceptualisations by demonstrating that sustainable person-centred cultures are not enacted solely through individual practitioner–resident interactions, but are underpinned by organisation-wide mechanisms that support, motivate, and empower all categories of staff through education and training in person-centred practices. Themes relating to staff education and training, leadership support, teamwork, and organisational commitment align particularly with the prerequisites and care environment constructs of the framework, which emphasise professional competence, interpersonal skills, supportive organisational systems, effective staff relationships, and shared decision-making. This highlights the importance of moving beyond role-specific or profession-specific approaches towards collective organisational enactment of person-centred care.

Similarly, themes relating to autonomy, individualised care planning, relational communication, meaningful engagement, and social connection correspond with the person-centred processes component of the framework, particularly engagement, sympathetic presence, working with residents’ beliefs and values, and shared decision-making. These processes contributed to outcomes such as emotional wellbeing, fulfilment, autonomy, and reduced depressive symptoms among older persons in nursing homes.

Similarly, this review refines Person–Environment Fit Theory by identifying specific mechanisms through which the physical, social, and organisational environment supports the health and wellbeing of older persons. Programme Theories relating to environmental adaptations, meaningful activities, social participation, relational continuity, and organisational flexibility align with the theory’s emphasis on congruence between individuals and their environments in supporting wellbeing and functioning. The findings illustrate that environmental adaptations function not only to enhance accessibility, but also as motivational and enabling contexts that support older persons to preserve functional abilities, maintain independence, and remain socially and psychologically engaged. In doing so, the review strengthens theoretical understanding of how environmental, relational, and organisational factors interact to influence mental health outcomes in nursing home settings.

### 5.3 Implications for practice and policy

The findings of this review have important implications for nursing home practice and policy. First, the review indicates that education and training of all cadres of staff in person-centred practices, cultural competence, and person-centred communication should be prioritised as a core organisational strategy rather than as an adjunct to care delivery. The programme theories demonstrate that such education supports staff confidence, motivation, and responsiveness to older persons’ needs, thereby enabling the consistent enactment of person-centred cultures across nursing home settings. Policymakers and nursing home administrators should therefore invest in sustained, organisation-wide staff development initiatives that embed person-centred principles across all levels of care delivery and management.

Second, the findings highlight the importance of collaborative care planning and effective communication among staff, older persons, and families. Where care plans reflecting the preferences, needs, and choices of older persons are developed collaboratively and actively communicated across staff teams, collective staff behaviour is supported and staff are empowered to deliver individualised care. These processes enhance older persons’ autonomy and dignity and contribute to improved mental health outcomes. Nursing home management should therefore prioritise teamwork, shared communication structures, and the allocation of adequate resources to ensure that care plans are meaningful, actionable, and consistently implemented in practice.

Third, the review underscores the role of environmental adaptations, organisational commitment, leadership support, and staff relationships in supporting independence, engagement, and psychological wellbeing among older persons. The findings suggest that environmental design, organisational flexibility, innovative practices, and leadership support are critical to enabling older persons to preserve functional abilities, maintain independence, and remain socially engaged, including during periods of heightened restriction such as infection outbreaks. There is therefore a need for organisational commitment to adequate staffing levels, appropriate material resources, and environmental modifications that explicitly support the mental health and wellbeing of older persons in nursing homes.

Taken together, these implications suggest that person-centred cultures should be positioned as a strategic organisational priority, supported through aligned policies, leadership practices, workforce development, and environmental design. Such alignment is essential to creating the conditions under which person-centred care can meaningfully contribute to the prevention and amelioration of depression among older persons in nursing home settings.

### 5.4 Implications for future research

The refined programme theories developed through this review provide a theoretically informed foundation for future empirical inquiry. In the next phase of the wider doctoral study, these programme theories will be tested through realist evaluation, using empirical data to examine their explanatory power and transferability across nursing home contexts. Through this process, further refinement, confirmation, or refutation of the programme theories may occur, and additional theories may be generated.

Future research should also examine how these programme theories operate across diverse cultural, organisational, and policy contexts, and how variations in staffing models, leadership approaches, and environmental design influence the activation of key mechanisms. Longitudinal realist evaluations may be particularly valuable in exploring how person-centred cultures are sustained over time and how their mental health impacts evolve in response to organisational change. Together, these implications for practice, policy, and research reflect the explanatory value of the programme theories developed in this review and provide a foundation for their application and testing in real-world nursing home contexts.

### 5.5 Strengths and limitations of the findings

**Strengths**

This review adopted a robust realist design, incorporating input from expert and local reference panels. This approach enhanced the contextual relevance of the findings and ensured alignment with current healthcare priorities and practice realities. The use of realist methodology enabled the synthesis of diverse evidence sources and supported the development of explanatory programme theories that move beyond surface-level associations to explain how person-centred cultures influence depression among older persons in nursing homes.

**Limitations**

The literature search for this review was limited to studies published in the English language and to literature focused on nursing homes as operationalised for this review. While the exclusion of non-English publications in systematic reviews of interventions has been reported to have no effect on overall conclusions (Teeling et al., 2021), this nonetheless represents a limitation. In addition, publication bias may have influenced the findings, as the context–mechanism–outcome configurations were derived primarily from peer-reviewed literature.

In addition, the inclusion of expert panel from multiple countries provided diverse professional and cultural perspectives that contributed to the refinement and interpretation of the programme theories. However, we acknowledge that the geographic and cultural backgrounds of the expert panel may not have fully reflected the diversity of settings represented within the included studies. This therefore represents a potential limitation of the review, particularly given that understandings and implementation of person-centred cultures, family involvement, communication practices, and depression care may vary across sociocultural and organisational contexts.

It is also important to note that the context, mechanism, and outcome configurations were inferred through realist interpretation of the literature and may not always be explicitly articulated within primary studies. Consequently, the proposed mechanisms remain theoretical and require further empirical testing. This will be addressed in Phase Three of the wider study through realist evaluation, which will examine the transferability and explanatory value of the programme theories across real-world nursing home contexts.

## 6 Conclusion

This paper has presented Phase Two of a wider doctoral study, translating realist synthesis findings into explanatory programme theories that inform practice, policy, and future research. Through a realist synthesis, six data-driven programme theories were developed that explain how organisational, relational, environmental, and individual mechanisms interact within nursing homes to influence the management of depression among older persons.

Together, these findings provide actionable, theory-informed insights for practice, policy, and future research, demonstrating that organisation-wide adoption of person-centred cultures is central to addressing depression among older persons in nursing home settings and to promoting their psychological wellbeing.

## Availability of data and materials

The datasets used and/or analysed during the current study are available from the corresponding author upon reasonable request.

## Funding statement

No specific funding was received towards this research work.

## Declaration of generative AI and AI-assisted technologies in the writing process

We declare that generative AI was not used in writing of this manuscript.

## Appendices: Supplementary data

Appendix A: RAMESSES I Publication standards.

Appendix B: Initial programme theories.

Appendix C: PubMed Search strategy, adapted across other databases.

Appendix D: Inclusion/exclusion/ relevance, richness and rigor criteria.

Appendix E: Sample of a data extraction form.

Appendix F: Illustrative table of synthesis of initial programme theories into programme theories.

Appendix G: Study Characteristics and Relevant Components of Person-centred Cultures of the Included Studies.

## CRediT authorship contribution statement

**Tope Omisore:** Writing – review & editing, Writing – original draft, Visualization, Validation, Resources, Project administration, Methodology, Investigation, Formal analysis, Data curation, Conceptualization. **Seán Paul Teeling:** Writing – review & editing, Writing – original draft, Validation, Supervision, Methodology, Formal analysis, Data curation, Conceptualization. **Timmy Frawley:** Writing – review & editing, Writing – original draft, Validation, Supervision, Methodology, Formal analysis, Conceptualization.

## Declaration of competing interest

We declare we have no known competing interest that could have influenced the findings reported in this paper.

## Appendix Supplementary materials

Image, application 1
