# Supplementary material for: A realist review of the contribution of person-centred cultures to the management of depression among older persons in nursing homes
Source: Int J Nurs Stud Adv. 2026 Jul 3;11:100618. doi: 10.1016/j.ijnsa.2026.100618 (PMC13418227; doi:10.1016/j.ijnsa.2026.100618)
Supplement: Supplementary file 1 [file mmc1.docx]

**Appendix A: RAMESES Publication Standards Checklist (Wong et al., 2013)**

| **Section** | **Publication Standard** | **Location** |
| --- | --- | --- |
| 1. Title | Review identified as a realist review in the title | Title, lines 1-2 |
| 2. Abstract | Contains study background, objectives, search strategy, methods of selection, appraisal, and synthesis, main results, and implications for practice | Abstract |
| 3. Rationale | The review is needed to explain whether or not, how, why and in what contexts person-centred cultures contribute to the management of depression among older persons in nursing homes | Introduction, lines 67-78 |
| 4. Objectives and focus | Review question:  Do person-centred cultures contribute to the management of depression among older persons in nursing homes, how, in what circumstances, and why?  Objectives: 1) To determine whether person-centred cultures contribute or not to the management of depression among older persons in nursing homes, how and in what contexts. 2) To determine the facilitators and barriers of developing person-centred cultures that may contribute to the management of depression among older persons in nursing homes  3) To explore in what contexts, do specific mechanisms facilitate or hinder nursing home staff’s understanding of the development of person-centred cultures, and how does this understanding influence the management of depressive symptoms among older persons  Focus:  Management of depression among older persons in nursing homes | Methods, lines 140-149 |
| 5. Changes in the review process | There was no change in the review process | Methods: not applicable |
| 6. Rationale for using realist synthesis | To explain how and why person-centred cultures contribute to the management of depression among older persons in nursing homes, given their complex nature and mixed outcomes reported. | Introduction and design, lines 79-132 |
| 7. Scoping the literature | Developing the candidate program theories explained | Clarifying scope of study, lines 183-188. |
| 8. Searching processes | Formal searches explained | Searching for evidence, lines 202-246 |
| 9. Selection and appraisal of documents | Screening and quality appraisal, including criteria explained and justified | Search for and appraise evidence, lines 227-246 |
| 10. Data extraction | Data extraction process explained | Extraction and synthesis of findings, lines 271-290 |
| 11. Analysis and synthesis processes | Analysis and synthesis processes explained | Extraction and synthesis, lines 248-267 |
| 12. Document flow diagram | A flow of sources diagram was provided, including formal search results | Figure 3, lines 288-289 |
| 13. Document characteristics | Characteristics of papers included in the formal complementary searches are provided | Documents characteristics, lines 291-302 |
| 14. Main findings | Key findings were provided with a focus on the refinement of the program theories. | Findings, lines 313-442 |
| 15. Summary of findings | A summary of findings was provided, considering the review question, objectives, focus, and audience | Discussion, lines 444-545 |
| 16. Strengths, limitations, and future directions | Strengths, limitations, future directions, with a discussion of the overall strength of evidence supporting the program theories | Discussion, lines 660-680 |
| 17. Comparison with existing literature | Comparison with existing literature and substantive theories is provided. | Discussion, lines 444-545 |
| 18. Conclusion and recommendations | A conclusion is provided, along with recommendations for policy and practice for stakeholders in nursing homes | Discussion and Conclusion, lines 444-692 |
| 19. Funding | Funding sources and conflicts of interest are outlined | Line 698, lines 711-712 |

**Appendix B: Initial Programme Theories (IPT)**

**IPT 1:** Where staff members are educated and trained in practice of person-centred approaches**(C1)** and actively use person-centred practice in the care of older persons with depressive symptoms**(C2)** in a nursing home that has person-centred practice champions**(C3)**, the champions support the development of person-centred cultures**(M1)** and support education and training in person-centred approaches for other staff within their practice through practice leadership**(M2)** and social modelling**(M3**),leading to a cohort of staff who facilitate the early detection and amelioration of depressive symptoms exhibited by older persons**(O1)(Bandura, 2003;Smith et al., 2013; Ballard et al., 2018;Kuo et al., 2019; Bonawitz et al., 2020; Kong et al., 2022).**

**IPT 2:** In a nursing home where there is a culture of respect for older persons, respect for their choice and autonomy**(C1)** and where there are contexts of low staff turnover**(C2),** high staff to older persons ratios**(C3**), effective communication among older persons, staff and families of older persons**(C4**) and an organisational commitment to actioning care plans(**C5)**, the mechanisms of person-centred care planning**(M1),** staff maximising older persons’ contact time(**M2**) and timely communication of older persons’ needs**(M3),** result in older persons experiencing person-oriented rather than task oriented care**(01),** intrinsic motivation**(O2)** and potential amelioration of depressive symptoms(**O3**)(Ryan and Deci, 2000; Hunter et al., 2016; Chao, 2019; Griffiths et al., 2019; Backman et al., 2020; Dys et al., 2022; Kong et al., 2022).

**IPT 3:** In a nursing home where there is a culture of promoting social engagement for older persons (**C1)** and there is a recognition by staff that older persons may have different levels of cognitive abilities(**C2**) in a context of organisational preparedness for the restrictive nature of socialisation during potential infection outbreak (**C3**), older persons are motivated to participate via the mechanism of individualised activities**(M1**) and alternative means of social engagement during an outbreak of infection(**M2)** resulting in older persons experiencing positive reinforcements(**O1),** enjoyable meaningful activities (**O2**), authentic rapport**(O3**), and psychological relatedness(**04)**,thereby reducing the potential of development of depressive symptoms(Frijters et al., 2011; Kang, 2012; Lewinsohn, 1974; Ryan and Deci, 2000).

**IPT 4:** In a nursing home that has the culture of promoting the independence of older persons(**C1)** where there are identifiable person-centred practice champions(**C2**) (PCP), older persons are supported in maintaining their independence by the mechanisms of an available and appropriately staffed MDT (**M1)** ,an appropriate built environment (e.g. provision of hand rails) **(M2**) and the support of PCP champions**(M3**), older persons experience social engagement(**O1**), autonomy**(O2**) and potential amelioration of depressive symptoms**(O3)(Kang , 2012; Chenoweth et al., 2018; Petrosvky et al., 2019; Bonawitz et al., 2020)**

**Appendix C: Search strategy for PubMed**

| **Item** | **Description** |
| --- | --- |
| **Database** | PubMed (MEDLINE) |
| **Platform** | National Library of Medicine |
| **Date searched** | 16/12/2024 |
| **Search strategy name** | PubMed PICO-based strategy |
| **Search fields** | Title/Abstract and MeSH terms |
| **Limits applied** | English language; publication years 1990–2024 |
| **Search concepts combined using** | AND |
| **Search concept 1 (P1: Population – Older adults)** | Older* OR Elder* OR Geriatric* OR “Late life” OR Senior* OR “Late on-set” OR Old OR Aging OR Aged* OR Senescence OR Senesence OR“Aged”[Mesh] OR “Frail Elderly”[Mesh] OR “Aged, 80 and over”[Mesh] |
| **Search concept 2 (P2: Setting – Nursing homes)** | “Nursing home*” OR “Homes for the aged” OR “Old age home*” OR “Care home*” OR “Old people’s home*” OR “Residential care facilit*” OR “Residential aged care facilit*” OR “Senior housing” OR “Residential facilit*” OR“Nursing Homes”[Mesh] OR “Intermediate Care Facilities”[Mesh] OR “Long-Term Care”[Mesh] OR “Residential Facilities”[Mesh] OR “Homes for the Aged”[Mesh] |
| **Search concept 3 (I: Intervention/Exposure – Person-centredness, culture, practice)** | People-centredness OR “People centredness” OR People-centeredness OR “People centeredness” OR Patient-centredness OR Patient-centeredness OR “Patient centredness” OR “Patient centeredness” OR“Person-centred culture*” OR “Person-centered culture*” OR “Person centred culture*” OR “Person centered culture*” OR “Patient-centred culture*” OR “Patient-centered culture*” OR “Patient centred culture*” OR “Patient centered culture*” OR“Organisational culture*” OR “Organizational culture*” OR “Eden alternative*” OR “Culture change” OR Culture-change OR“Person-centred approach*” OR “Person-centered approach*” OR “Person centred approach*” OR “Person centered approach*” OR “Patient-centred approach*” OR “Patient-centered approach*” OR “Patient centred approach*” OR “Patient centered approach*” OR“Person-centred practice*” OR “Person-centered practice*” OR “Person centred practice*” OR “Person centered practice*” OR “Patient-centred practice*” OR “Patient-centered practice*” OR“Patient-focussed care*” OR “Patient focussed care” OR “Person focussed care” OR “Person-focussed care” OR “Patient centred nursing” OR “Patient-centered nursing” OR “Patient centered nursing” OR “Person centred nursing” OR “Person-centered nursing” OR “Person centered nursing” OR “Person-centred nursing” OR“Social engagement*” OR “Person-centred care” OR “Person centred care” OR “Person-centered care” OR “Person centered care” OR “Patient-centred care” OR “Patient centred care” OR “Patient-centered care” OR “Patient centered care” OR“Person care plan*” OR “Patient care plan*” OR “Meaningful activit*” OR “Patient focus care” OR “Person focus care” OR “Dementia care mapping” OR Individualis* OR Individualiz* OR Personaliz* OR Personaliz* OR“Relationship centred care” OR “Relationship centered care” OR “Relationship-centred care” OR “Relationship-centered care” OR PCC OR DCM OR “Client-centred care” OR “Client-centered care” OR “Client centred care” OR “Client centered care” OR“Culturally competent care” OR “Culturally-competent care” OR “Quality care” OR “Care quality” OR Deinstitutionalis* OR Deinstitutionaliz* OR“Nursing care plan*” OR “Staff training” OR “Staff development” OR “Staff education” OR “Inservice training” OR “On the job training” OR “On-the-job-training” OR “Professional development” OR “Continuing education” OR “Personnel development” OR“Patient-Centered Care”[Mesh] OR “Organizational Culture”[Mesh] OR “Social Participation”[Mesh] OR “Culturally Competent Care”[Mesh] OR “Staff Development”[Mesh] OR “Inservice Training”[Mesh] |
| **Search concept 4 (O: Outcomes – Psychological and motivational outcomes)** | Depress* OR Anhedonia OR Melanchol* OR “Intrinsic motivation” OR “Authentic rapport” OR Reinforcement* OR “Psychological relatedness” OR Autonomy OR “Personal autonomy” OR “Self-determination” OR “Self determination” OR“Depression”[Mesh] OR “Depressive Disorder”[Mesh:NoExp] OR “Depressive Disorder, Major”[Mesh] OR “Dysthymic Disorder”[Mesh] OR “Anhedonia”[Mesh] OR “Personal Autonomy”[Mesh] OR “Reinforcement, Psychology”[Mesh] |
| **Combination logic** | P1 AND P2 AND I AND O |
| **Number of records retrieved** | 436 |

**Appendix D: Relevance, Richness and Rigor Criteria (Dada et al., 2023)**

| **Domain** | **Criteria** |
| --- | --- |
| **Relevance** | **Include:** Studies involving older adults (≥60 years) living in nursing homes; empirical studies of any design producing data relevant to programme theory development or testing (excluding systematic reviews and meta-analyses); published and unpublished sources (including grey literature); studies published between 1990 and 2024.  **Exclude:** Studies conducted outside nursing home settings; studies unable to contribute to programme theory development or testing; non-English language publications; studies outside the specified timeframe. |
| **Richness** | **High:** Data provided rich explanatory detail enabling multiple contributions to programme theory development, including contexts, mechanisms and outcomes.  **Moderate:** Data enabled one or two clear contributions to programme theory development.  **Low:** Data made a limited contribution with minimal explanatory detail.  **None:** Data were relevant to the topic but did not contribute to programme theory development. |
| **Rigour** | In line with RAMESES guidance, rigour was assessed to determine the trustworthiness and interpretive weight of data rather than as a basis for exclusion. Assessment considered methodological credibility, corroboration across sources, and the coherence and plausibility of developing programme theories. Expert panel input supported rigour judgements. No documents were excluded on the basis of rigour alone. |

**Appendix E. Sample Customised Realist Data Extraction Form**

**Study Identification**

| **Field** | **Details** |
| --- | --- |
| Covidence record number | 2138 |
| Reviewer(s) | TO / SPT |
| Title | Person-centred Care Transformation in a Community Nursing Unit for Residents with Dementia |
| First author (year) | Ho et al. (2021) |
| Project / intervention name | Not specified |
| Study objectives | To describe the PCC model adopted by a nursing home using a logic model and to evaluate outcomes related to residents’ wellbeing, care quality, and staff attrition. |
| PCC interventions | Leadership training; activity-centred care; changes to the physical environment; staff training and education in PCC. |
| Companion papers / grey literature | None identified |

**Appraisal Assessment (completed at end of extraction)**

| **Domain** | **Rating** |
| --- | --- |
| Usefulness and relevance | Relevant |
| Richness | High |
| Rigour | High |

**A. Summary of the Paper**

| **Aspect** | **Description** |
| --- | --- |
| Study type | Quantitative, peer-reviewed |
| Design | Observational cohort study |
| Setting | 30-bed assisted living facility for male persons with dementia |
| Methods | Dementia Care Mapping used to assess resident wellbeing; staff attrition measured pre- and post-PCC implementation |
| Data source | Quantitative |

**B. What Is Important About This Paper?**

(Explanatory contributions to programme theory)

| **CMO** | **Context (C)** | **Mechanism (M)** | **Outcome (O)** | **Relevant IPT** |
| --- | --- | --- | --- | --- |
| CMO 1 | Staff at all organisational levels trained in PCC | Staff feel empowered to innovate care | Enhanced autonomy and wellbeing; reduced depressive symptoms | IPT 1 |
| CMO 2 | PCC education, in-service training, practitioner-leaders | Mentorship, motivation, empathy, adaptive practice | Amelioration of depressive symptoms | IPT 1 |
| CMO 3 | Culture of social engagement and meaningful activity | Meaningful occupation | Prevention of disability; reduced depressive symptoms | IPT 6 |
| CMO 4 | Culture promoting independence and a home-like environment | Inclusivity; enhanced autonomy in ADLs | Maintained personhood; reduced depressive symptoms | IPT 4 |
| CMO 5 | Respect for choice and preferences; use of living well plans | Deep understanding of residents; staff empowerment | Personalised care; upheld identity and dignity; prevention of depression | IPT 2 |

**Additional Information and Key Excerpts From the Text**

| **Extract** |
| --- |
| “Embracing PCC at every level of the organization empowered staff to re-invent care with the new priorities of enhancing residents’ well-being, autonomy, and independence.” |
| “Both classroom and on-the-job training afforded opportunities for role-modelling as an essential component of in-house learning.” |
| “The incorporation of meaningful activities into the residents’ routines promotes their well-being and prevents disability.” |
| “An inclusive and home-like environment helped to maintain residents’ personhood, facilitating enhanced physical and overall well-being.” |
| “The use of living well plans provided a deep understanding of each resident and afforded personalised care that upheld identity and dignity.” |

**D. Unintended Positive or Negative Impacts and Mechanisms**

| **Item** | **Description** |
| --- | --- |
| Unintended positive impacts | None reported |
| Unintended negative impacts | None reported |
| Mechanism explanation | Not applicable |

**E. Questions for the First Author and Research Partners**

| **Item** | **Details** |
| --- | --- |
| Questions to strengthen programme theory | None identified |

**Appendix F. Example of the Synthesis and Refinement of Initial Programme Theories into Programme Theories**

**Initial Programme Theory (IPT ONE):**

**Staff Education and Training in Person-centred Approaches**

Where staff members are educated and trained in the practice of person-centred approaches (C1) and actively use person-centred practices in the care of older persons with depressive symptoms (C2) in nursing homes that have person-centred practice champions (C3), these champions support the development of person-centred cultures (M1) and facilitate education and training through practice leadership (M2) and social modelling (M3), leading to staff who facilitate the early detection and amelioration of depressive symptoms in older persons (O1).

**Mapping Extracted Data to IPT ONE**

| **Study (Author, Year)** | **CMO** | **Covidence ID** | **Context (C)** | **Mechanisms (M)** | **Outcomes (O)** | **Explanatory Contribution** | **Judgement** |
| --- | --- | --- | --- | --- | --- | --- | --- |
| Ho et al. (2021) | CMO 1 | 2138 | C1 | M4 | O4, O6 | Emphasises staff training across all cadres in person-centred approaches, empowering staff to innovate care that upholds autonomy and wellbeing, resulting in amelioration of depressive symptoms. | Refine 🔄 |
| Ho et al. (2021) | CMO 2 | 2138 | C1, C2 | M1 | O6 | Highlights the role of practice leaders in supporting staff adaptation to older persons’ changing needs, leading to reduced depressive symptoms. | Refine 🔄 |
| Wright (2010) | CMO 8 | 5482 | C1 | M2, M4 | O5, O6 | Demonstrates how education and training in person-centred and depression care increase staff confidence and value placed on social engagement, supporting early identification and amelioration of depressive symptoms. | Refine 🔄 |
| Chenoweth et al. (2014) | CMO 19 | 886 | C1, C2 | M1, M2, M3 | O1, O2, O3 | Shows how training, environmental adaptation, leadership support and champions preserve independence, improve social functioning and reduce depressive symptoms. | Refine 🔄 |
| Schweighart et al. (2022) | CMO 16 | 4445 | C1, C3 | M3 | O2, O3, O6 | Emphasises education in communication skills and manageable workloads, motivating staff to engage positively with older persons and improve relationships, leading to amelioration of depressive symptoms. | Refine 🔄 |

**Resulting Programme Theory (PT ONE)**

**PT1 – Staff Education and Leadership Support for Person-centred Care**

In nursing homes where staff at all levels are educated and trained in person-centred approaches, including care for depression (C1), where there are identifiable person-centred practice champions or practitioner-leaders who support a culture of continuous development (C2), and where staff workloads are manageable (C3), mechanisms of leadership support (including mentorship, motivation and role modelling) (M1), alongside increased staff confidence (M2), motivation (M3), innovation and empowerment (M4) to adapt to older persons’ changing needs, lead to a culture of embedded person-centred practices (O1), stronger staff–older person relationships and communication (O2), staff taking older persons’ needs seriously (O3), upholding autonomy (O4), and facilitating both early detection (O5) and amelioration of depressive symptoms (O6).

**Appendix G: Study Characteristics and Relevant Components of Person-centred Cultures of the Included Studies**

| **First Author and Year** | **Objectives and method** | **Location** | **Person-centred Culture Component** |
| --- | --- | --- | --- |
| Ho et al (2021) | Study described the model of Person-Centred Care (PCC) adopted by a nursing home, Apex  Harmony Lodge (AHL), with a logic model and evaluate outcomes on residents’ well-being, care quality, and staff attrition by comparing pre-PCC initiation (2015) to post-implementation (2016). | Singapore | Organisation wide-staff training and education in person-centred practice, provision of meaningful activities, use of living well plan (care plans), environmental adaptations and family involvement |
| Knippenberget et al (2023) | The study aimed to identify and structure potential informal antidepressant strategies that can be used  in daily practice for nursing home residents alongside formal treatments. | Netherland | Environmental adaptations, provision of meaningful activities and having social connections. |
| Schweighart et al (2022) | The study aimed to  examine the needs of nursing home residents with depressive symptoms and the communication of  those needs. Used qualitative methods. | Germany | Centred on education/ training in PC approaches especially communication skills and the importance of manageable workloads for staff |
| Duan et al (2024) | The study explored Nursing Home Resident Preferences for Daily Care and  Activities: A Latent Class Analysis of National Data | United States of America | Person-centred care plans and family involvement in care of older persons and individualised activities |
| Ditcher et al (2020) | COVID-19: it is time to balance infection management and  person-centred care to maintain mental health of people  living in German nursing homes. A commentary |  | Social connections during infection outbreaks |
| Lindner et al (2023) | The study aimed to evaluate nursing staff and manager perceptions of the opportunities to perform person-centred care during the COVID-19 pandemic. A quantitative study. | Sweden | Leadership support for social connections for older adults during infection outbreaks |
| Hunter et al (2016) | This study aimed to investigate the association of personal and  organizational environmental characteristics with self-reported person-centred behaviours in long-term residential care  settings. Quantitative study. | Canada | Environmental adaptations, autonomy and social engagement. |
| van Loon et al (2023) | The study aimed to gain a deeper insight into the development and implementation of organisational policies aimed to enhance the autonomy of older adults with physical  impairments. A qualitative descriptive design was used. | Netherlands | Organisational policies and autonomy of older persons. |
| Kussmaul and Tucker (2020) | This study aimed to understand how  people in different positions experience person-centred care by exploring how various  stakeholders (residents, family members, direct care staff, managers) defined selected person-  centred care practices (consistent assignment, meal choice, waking/bedtime, and bathing) and  compared their perspectives. | United State of America | Staff training, leadership and organisational commitment to person-centred cultures |
| Murphy (2007) | The aim of this research was to determine the factors that facilitate or hinder  high quality nursing care for older people in long-term care settings in Ireland. A mixed method study | Ireland | Ward managers training in person-centred care and autonomy for older persons |
| Backman et al (2024) | The aim of this study was to explore factors characterizing high and low person-centred nursing home units, with focus on leadership, staff, resident and facility variables. | Sweden | Staff training and education in person-centred practice |
| Backman et al (2020) | To explore how managers describe leading towards person‐  centred care in Swedish nursing homes. | Sweden | Use of person-centred care plans and importance of leadership in person-centred care implementation |
| Backman et al (2021) | Explored factors characterizing high and low person-centred nursing home units, with focus on leadership, staff, resident and facility variables. | Sweden | Environmental adaptations |
| Wikström and Emilsson (2014) | This article discusses older people’s experiences with and the opportunities for autonomy in institution-based housing. Through focus group interviews and observations, the daily lives of residents at two nursing homes in Sweden were studied through a comparative approach using the theoretical framework of organizational culture. Qualitative study. | Sweden | Autonomy and importance of shared valued in promoting person-centred cultures. |
| Cohen-Mansfield (2008) | The paper seeks to operationalize  those aspects of the nursing home practice style that can be improved in nursing homes. A piece | Not applicable | Staff training and organisational commitment to person-centred practices |
| Hung et al (2016) | This qualitative study evaluated the effect of dining room physical  environmental changes on staff practices and residents’ mealtime experiences  in two units of a long-term care facility in Edmonton, Canada. | Canada | Environmental adaptations and leadership support in facilitating autonomy of older persons |
| Sjögren et al (2013) | To report a study of the relationship between person-centred care and  ability to perform activities of daily living, quality of life, levels of pain,  depressive symptoms, and agitated behaviours among residents with dementia in  residential care facilities. Quantitative study | Sweden | Independence-supportive staff practices |
| Lindsey et al (2019) | The study identifies and describes specific autonomy-supportive  techniques nurse assistants used in a LTC setting that was in the process of  shifting toward a PCC model of care using observation and interviews | United State of America | Autonomy-supportive staff practices |
| Potter et al (2018) | This study explores the relationship between the physical environ-  ment and depressive symptoms of older people living in care homes. Mixed methods | United Kingdom | Environmental adaptations |
| Shenell et al (2020) | The aim of the study was to develop a model that facilitates self-determination in residential care. Focus group. | Sweden | Autonomy, independence -supportive staff practices and self-determination for older persons |
| Chenoweth et al (2014) | PerCEN: a cluster randomized controlled trial of  person-centered residential care and environment  for people with dementia | Australia | Environmental adaptations, staff training and leadership support for person-centred care |
| Lou et al (2013) | This study examines the relationship between social engagement and depressive symptoms and changes in social  engagement and depressive symptoms among Chinese residents of long-term care facilities over 6 years.  Design and methods: a latent growth model was used to analyse six waves of data collected using the Resident  Assessment Instrument Minimum Data Set 2.0 in the Hong Kong | Hong Kong | Social engagement with older persons |
| Duan et al (2020) | Unmet and Unimportant Preferences Among Nursing Home  Residents: What Are Key Resident and Facility Factors? A longitudinal study | United State of America | Promoting choice and autonomy of older persons |
| Lyne et al (2006) | Analysis of a care planning intervention for reducing depression  in older people in residential care: quasi experimental study | United Kingdom | Care planning and staff-older person relationship |
| Yoon (2018) | The purpose of this study was to examine whether a perceived person-centred nursing home  environment has a direct relationship with nursing home adjustment and life satisfaction, and whether  a perceived person-centred nursing home environment has an indirect relationship with life satisfaction  through improved nursing home adjustment. A quantitative study. | United States of America. | Person-centred nursing home environment and staff training in person-centred practices |
| Xiao et al (2023) | To explore and compare staff perceived challenges and facilita-  tors in supporting resident self-determination in ethno-specific and mainstream nurs-  ing homes. Qualitative | Australia | This paper emphasizes the importance of training in cultural competence and team work, and family involvement in the development of person-centred care plans |
| Haugan et al (2023) | To test the effects of nurse–patient interaction on anxiety and depression among cognitively intact  nursing home patients. Quantitative | Norway | Focuses on social engagement for older persons |
| Carcavilla-González et al (2024) | The objective of this study was to examine the benefits of a multicomponent person-centred support initiative for older people living in nursing homes. Randomised controlled trial. | Spain | Focuses on the importance of personalized engagement with older persons |
| Wright (2010) | Prevention and treatment of  depression in care homes. A commentary | Not applicable | This paper focuses on the importance of staff training and social engagement for older persons. |
| Erlandsson et al (2023) | The aim of this article is to explore how nursing home staff and managers perceive  the participation of older residents and what the implications are for  residents’ involvement in decision-making in everyday life. Interviews  and future workshops were carried out with staff and managers in two  Swedish nursing homes. | Sweden | Organisational commitment to person-centred cultures |

References

Ballard, C., Corbett, A., Orrell, M., Williams, G., Moniz-Cook, E., Romeo, R., Woods, B., Garrod, L., Testad, I., Woodward-Carlton, B., Wenborn, J., Knapp, M. & Fossey, J. 2018. Impact Of Person-Centred Care Training And Person-Centred Activities On Quality Of Life, Agitation, And Antipsychotic Use In People With Dementia Living In Nursing Homes: A Cluster-Randomised Controlled Trial. Plos Medicine, 15, 1-18.

Bandura, A. 2003. Social Cognitive Theory For Personal And Social Change By Enabling Media. Entertainment-Education And Social Change. Routledge.

Bethell, J., Aelick, K., Babineau, J., Bretzlaff, M., Edwards, C., Gibson, J.-L., Hewitt Colborne, D., Iaboni, A., Lender, D., Schon, D. & Mcgilton, K. S. 2021. Social Connection In Long-Term Care Homes: A Scoping Review Of Published Research On The Mental Health Impacts And Potential Strategies During Covid-19. Journal Of The American Medical Directors Association, 22, 228-237.E25.

Backman, A., Sandman, P.-O. & Sköldunger, A. 2021. Characteristics Of Nursing Home Units With High Versus Low Levels Of Person-Centred Care In Relation To Leadership, Staff- Resident- And Facility Factors: Findings From Swenis, A Cross-Sectional Study In Sweden. Bmc Geriatrics, 21, 498.

.

Bonawitz, K., Wetmore, M., Heisler, M., Dalton, V. K., Damschroder, L. J., Forman, J., Allan, K. R. & Moniz, M. H. 2020. Champions In Context: Which Attributes Matter For Change Efforts In Healthcare? Implementation Science, 15, 62.

Chao, S.-F. 2019. Does Geriatric Nursing Staff Burnout Predict Well-Being Of Ltc Residents? Geriatric Nursing, 40, 360-366.

Chenoweth, L., Jessop, T., Harrison, F., Cations, M., Cook, J. & Brodaty, H. 2018. Critical Contextual Elements In Facilitating And Achieving Success With A Person-Centred Care Intervention To Support Antipsychotic Deprescribing For Older People

Dys, S., Tunalilar, O., Hasworth, S., Winfree, J. & White, D. L. 2022. Person-Centered Care Practices In Nursing Homes: Staff Perceptions And The Organizational Environment. Geriatric Nursing, 43, 188-196.

Frijters, D. H. M., Groenewegen, P. P., Ribbe, M. W., Wagner, C. & Van Beek, A. P. A. 2011. Social Engagement And Depressive Symptoms Of Elderly Residents With Dementia: A Cross-Sectional Study Of 37 Long-Term Care Units. International Psychogeriatrics, 23, 625-633.

Griffiths, A. W., Kelley, R., Garrod, L., Perfect, D., Robinson, O., Shoesmith, E., Mcdermid, J., Burnley, N. & Surr, C. A. 2019. Barriers And Facilitators To Implementing Dementia Care Mapping In Care Homes: Results From The Dcm™ Epic Trial Process Evaluation. Bmc Geriatrics, 19, 37.

Hunter, P. V., Hadjistavropoulos, T., Thorpe, L., Lix, L. M. & Malloy, D. C. 2016. The Influence Of Individual And Organizational Factors On Person-Centred Dementia Care. Aging & Mental Health, 20, 700-708.

Kang, H. 2012. Correlates Of Social Engagement In Nursing Home Residents With Dementia. Asian Nursing Research, 6, 75-81.

Kong, E.-H., Kim, H. & Kim, H. 2022. Nursing Home Staff's Perceptions Of Barriers And Needs In Implementing Person-Centred Care For People Living With Dementia: A Qualitative Study. Journal Of Clinical Nursing, 31, 1896-1906.

Kuo, C.-L., Wang, S.-Y., Tsai, C.-H., Pan, Y.-F. & Chuang, Y.-H. 2019. Nurses’ Perceptions Regarding Providing Psychological Care For Older Residents In Long-Term Care Facilities: A Qualitative Study. International Journal Of Older People Nursing, 14, E12242.

Lewinsohn, P. M. 1974. A Behavioral Approach To Depression. Essential Papers On Depression, 150-172.

Petrovsky, D. V., Sefcik, J. S., Hanlon, A. L., Lozano, A. J. & Cacchione, P. Z. 2019. Social Engagement, Cognition, Depression, And Comorbidity In Nursing Home Residents With Sensory Impairment. Research In Gerontological Nursing, 12, 217-226.

Ryan, R. M. & Deci, E. L. 2000. Self-Determination Theory And The Facilitation Of Intrinsic Motivation, Social Development, And Well-Being. American Psychologist, 55

Sjögren, K., Bergland, Å., Kirkevold, M., Lindkvist, M., Lood, Q., Sandman, P.-O., Vassbø, T. K. & Edvardsson, D. 2022. Effects Of A Person-Centred And Thriving-Promoting Intervention On Nursing Home Residents’ Experiences Of Thriving And Person-Centredness Of The Environment. Nursing Open, 9, 2117-2129.

Smith, M., Stolder, M. E., Jaggers, B., Liu, M. F. & Haedtke, C. 2013. Depression Training In Nursing Homes: Lessons Learned From A Pilot Study. Issues In Mental Health Nursing, 34, 90-102.

Carcavilla-González, N., José, G.-M. J., Rabaneda, B. R. & and Torres-Castro, S. 2025. Transforming Nursing Home Support: Person-Centered Pilot Effects. Activities, Adaptation & Aging, 49, 116-135. 10.1080/01924788.2024.2317031

Wright, K. 2010. Prevention and treatment of depression in care homes. Nursing & Residential Care, 12, 188-191, :10.12968/nrec.2010.12.4.47101

Ho, P., Cheong, R. C. Y., Ong, S. P., Fusek, C., Wee, S. L. & Yap, P. L. K. 2021. Person-Centred Care Transformation in a Nursing Home for Residents with Dementia. Dementia and Geriatric Cognitive Disorders Extra, 11, 1-9, 10.1159/000513069

Schweighart, R., Klemmt, M., Neuderth, S. & Teti, A. 2022. Needs and Needs Communication of Nursing Home Residents with Depressive Symptoms: A Qualitative Study. International Journal of Environmental Research and Public Health, 19, 10.3390/ijerph19063678

Chenoweth, L., Forbes, I., Fleming, R., King, M. T., Stein-Parbury, J., Luscombe, G., Kenny, P., Jeon, Y.-H., Haas, M. & Brodaty, H. 2014. PerCEN: a cluster randomized controlled trial of person-centered residential care and environment for people with dementia. International Psychogeriatrics, 26, 1147-1160, 10.1017/S1041610214000398

Cohen-Mansfield, J. & Parpura-Gill, A. 2008. Practice style in the nursing home: Dimensions for assessment and quality improvement. International Journal of Geriatric Psychiatry, 23, 376-386, 10.1002/gps.1888

Backman, A., Ahnlund, P., Sjögren, K., Lövheim, H., McGilton, K. S. & Edvardsson, D. 2020. Embodying person-centred being and doing: Leading towards person-centred care in nursing homes as narrated by managers. Journal of Clinical Nursing, 29, 172-183, 10.1111/jocn.15075

Xiao, L. D., Gregoric, C., Gordon, S., Ullah, S., Goodwin-Smith, I., Muir-Cochrane, E. & Blunt, S. 2023. Staff perceived challenges and facilitators in supporting resident self-determination in ethno-specific and mainstream nursing homes. Journal of Clinical Nursing, 32, 3630-3643, 10.1111/jocn.16440

Duan, Y., Shippee, T. P., Ng, W., Akosionu, O., Woodhouse, M., Chu, H., Ahluwalia, J. S., Gaugler, J. E., Virnig, B. A. & Bowblis, J. R. 2020. Unmet and Unimportant Preferences Among Nursing Home Residents: What Are Key Resident and Facility Factors? Journal of the American Medical Directors Association, 21, 1712-1717, 10.1016/j.jamda.2020.06.033

Erlandsson, S., Knutsson, O. & Schön, U.-K. 2023. Perceptions of participation: how nursing home staff and managers perceive and strive for participation of older residents. European Journal of Social Work, 26, 815-827, 10.1080/13691457.2022.2094345

Backman, A., Ahnlund, P., Lövheim, H. & Edvardsson, D. 2024. Nursing home managers' descriptions of multi-level barriers to leading person-centred care: A content analysis. International Journal of Older People Nursing, 19, e12581, 10.1111/opn.12581

Duan, Y., Ng, W., Bowblis, J. R., Akosionu, O. & Shippee, T. P. 2024. Nursing Home Resident Preferences for Daily Care and Activities: A Latent Class Analysis of National Data. Gerontologist, 64, 1-11, 10.1093/geront/gnad089

Haugan, G., Innstrand, S. T. & Moksnes, U. K. 2013. The effect of nurse-patient interaction on anxiety and depression in cognitively intact nursing home patients. Journal of Clinical Nursing (John Wiley & Sons, Inc.), 22, 2192-2205, 10.1111/jocn.12072

Schenell, R., Ozanne, A., Strang, S. & Henoch, I. 2020. To make and execute decisions throughout life: A person-centred model that facilitates self-determination in residential care, developed through participatory research. Applied Nursing Research, 55, N.PAG-N.PAG, 10.1016/j.apnr.2020.151318

Yoon, J. Y. 2018. Relationships among person-centered care, nursing home adjustment, and life satisfaction: A cross-sectional survey study. International Psychogeriatrics, 30, 1519-1530, 10.1017/S1041610218000194

Lyne, K. J., Moxon, S., Sinclair, I., Young, P., Kirk, C. & Ellison, S. 2006. Analysis of a care planning intervention for reducing depression in older people in residential care. Aging & Mental Health, 10, 394-403, 10.1080/13607860600638347

Lou, V. W., Chi, I., Kwan, C. W. & Leung, A. Y. 2013. Trajectories of social engagement and depressive symptoms among long-term care facility residents in Hong Kong. Age Ageing, 42, 215-22, 10.1093/ageing/afs159

Lindsey Jacobs, M., Lynn Snow, A., Allen, R. S., Hartmann, C. W., Dautovich, N. & Parmelee, P. A. 2019. Supporting autonomy in long-term care: Lessons from nursing assistants. Geriatr Nurs, 40, 129-137, 10.1016/j.gerinurse.2018.07.004

Potter, R., Sheehan, B., Cain, R., Griffin, J. & Jennings, P. A. 2018. The Impact of the Physical Environment on Depressive Symptoms of Older Residents Living in Care Homes: A Mixed Methods Study. Gerontologist, 58, 438-447, 10.1093/geront/gnx041

Sjögren, K., Lindkvist, M., Sandman, P. O., Zingmark, K. & Edvardsson, D. 2013. Person-centredness and its association with resident well-being in dementia care units. Journal of Advanced Nursing, 69, 2196-2206, 10.1111/jan.12085

Backman, A., Sandman, P.-O. & Sköldunger, A. 2021. Characteristics of nursing home units with high versus low levels of person-centred care in relation to leadership, staff- resident- and facility factors: findings from SWENIS, a cross-sectional study in Sweden. BMC Geriatrics, 21, 498, 10.1186/s12877-021-02434-0

Murphy, K. 2007. Nurses' perceptions of quality and the factors that affect quality care for older people living in long-term care settings in Ireland. Journal of Clinical Nursing, 16, 873-884, 10.1111/j.1365-2702.2006.01633.x

Kusmaul, N. & Tucker, G. G. 2020. Person-centered care in nursing homes: Many stakeholders, many perspectives. Journal of Gerontological Nursing, 46, 9-13, 10.3928/00989134-20200327-01

Hung, L., Chaudhury, H. & Rust, T. 2016. The Effect of Dining Room Physical Environmental Renovations on Person-Centered Care Practice and Residents' Dining Experiences in Long-Term Care Facilities. Journal of Applied Gerontology, 35, 1279-1301, 10.1177/0733464815574094

van Loon, J., Janssen, M., Janssen, B., de Rooij, I. & Luijkx, K. 2023. Developing a person-centred care environment aiming to enhance the autonomy of nursing home residents with physical impairments, a descriptive study. BMC Geriatrics, 23, 10.1186/s12877-023-04434-8

Wikström, E. & Emilsson, U. M. 2014. Autonomy and Control in Everyday Life in Care of O lder People in Nursing Homes. Journal of Housing for the Elderly, 28, 41-62, 10.1080/02763893.2013.858092

Dichter, M. N., Sander, M., Seismann-Petersen, S. & Köpke, S. 2020. COVID-19: it is time to balance infection management and person-centered care to maintain mental health of people living in German nursing homes. International psychogeriatrics, 32, 1157-1160, https://doi.org/10.1017/S1041610220000897

Lindner, H., Kihlgren, A. & Pejner, M. N. 2023. Person-centred care in nursing homes during the COVID-19 pandemic: a cross sectional study based on nursing staff and first-line managers’ self-reported outcomes. BMC Nursing, 22, 276, 10.1186/s12912-023-01437-z

Hunter, P. V., Hadjistavropoulos, T., Thorpe, L., Lix, L. M. & Malloy, D. C. 2016. The influence of individual and organizational factors on person-centred dementia care. Aging & Mental Health, 20, 700-708, 10.1080/13607863.2015.1056771

Knippenberg, I. A. H., Leontjevas, R., Stoyanov, S., Persoon, A., Verboon, P., Vermeulen, H., van Lankveld, J. J. D. M. & Gerritsen, D. L. 2023. Informal antidepressant strategies for nursing home residents: two group concept mapping studies. Aging & Mental Health, 27, 251-262, 10.1080/13607863.2022.2057427

Wong, G., Greenhalgh, T., Westhorp, G., Buckingham, J. & Pawson, R. 2013. RAMESES publication standards: realist syntheses. BMC Medicine, 11, 21, 10.1186/1741-7015-11-21.

Dada, S., Aivalli, P., De Brún, A., Barreix, M., Chelwa, N., Mutunga, Z., Vwalika, B. & Gilmore, B. 2023. Understanding communication in community engagement for maternal and newborn health programmes in low- and middle-income countries: a realist review. Health Policy and Planning, 38, 1079-1098, 10.1093/heapol/czad078
